# Evaluation of organizational culture in enterprises in the Czech Republic using OCAI

**DOI:** 10.3389/fpsyg.2023.1297041

**Published:** 2023-12-11

**Authors:** Miluše Balková, Tsolmon Jambal

**Affiliations:** Department of Human Resource Management (HRM), Faculty of Corporate Strategy, Institute of Technology and Business in Ceske Budejovice, Ceske Budejovice, Czechia

**Keywords:** organizational culture, OCAI method, competing value framework, HRM, company values

## Abstract

The goal of this work was to use the Organizational Culture Assessment Instrument (OCAI) diagnostic method to find out the existing and preferred organizational culture in companies in the Czech Republic and then to evaluate their dependence on the size of the organizations. Data were collected in 2019–2021 and evaluated using Microsoft Excel and IBM SPSS 24 statistical program, e.g., descriptive statistics tools, one-sample z-test, analysis of variance and *post hoc* test (Tukey’s honest significant difference - HSD). The research was conducted on a sample of 962 companies across the entire republic and fields. The results of the study show that clan culture prevails in the Czech Republic, which was dominant in all six dimensions. Enterprises in the Czech Republic have a mix of organizational cultures in the following order: (1) clan culture (31.72%), (2) hierarchical culture (25.46%), (3) market culture (21.5%), and (4) adhocratic culture (21.28%). However, with regard to the desired cultural mix, this order changes as follows: (1) clan (35.3%), (2) hierarchical (22.91%), (3) adhocratic (22.63%), and (4) market culture (19.17%). Furthermore, it was found that a statistically significant difference was observed in the assessment of organizational culture depending on the size of the organization in the Czech Republic. A limitation of the research could be the unequal number of respondents in 2020 and 2021, which does not allow comparing differences in the time period. This work can serve as a comparative basis of organizational culture with another national culture.

## 1 Introduction

The business environment is a space of endless changes, to which business entities must respond in a timely and appropriate manner in order to maintain their competitiveness and sustainability. In order for entities to be able to fulfill their visions, they need to establish appropriate strategies and create an internal environment, and an appropriate organizational (further also corporate) culture that supports innovative thinking, teamwork, as well as employee discipline, and loyalty.

Organizational (corporate) culture creates an internal environment and at the same time is influenced by the external environment, helps managers initiate company activities, manage employee behavior, and build the company’s reputation on the market. At the same time, it is created, influenced, and completed by the managers themselves. In the light of different definitions of organizational culture, each organization has its own unique culture with its own values, shared attributes, signs, norms of behavior, and common experiences of the members of the organization.

Corporate culture is one of the factors that influence employee behavior and corporate performance (Flynn, 2022). The success of a business largely depends on its values, norms, rules, patterns of behavior, and rituals (Lorincová et al., 2016). As a result of constant market changes, pressure on competitiveness, and the search for innovation, it is necessary that the corporate culture also continuously changes and adapts (Miller, 2021). Effective company management requires a company culture to be examined, measured, and compared. Various diagnostic methods are used for this purpose. Organizational culture is studied for its importance in influencing workplace behavior, cognitive function, and outcomes of individuals and work groups (Heritage et al., 2014). If a company does not know the real state of its organizational culture, it is unable to evaluate whether this culture meets its needs (Dobrin et al., 2021) or whether its employees are satisfied (Van Huy et al., 2020). Research on organizational culture is essential from the perspective of firm performance, organizational change, knowledge management, and innovation (Zbieg et al., 2017; Bendak et al., 2020; Kim and Lee, 2020). Lorincová et al. (2022) state that a positive company culture can contribute to the further strategic development of companies and their successful operation on the market.

Since the organizational culture is constantly adapting and changing, as a result of modernization, innovation, competitiveness in the market, and the direction in which the given company wants to go, it is necessary regularly to examine, measure, and compare it. As a result, it is subsequently possible to increase the level of organizational culture and possibly adapt it to the conditions of the organization. The aim of the article is to evaluate the current state of the organizational culture of companies in the Czech Republic and subsequently to evaluate their dependence on the size of the companies.

## 2 Literature review

Organizational culture is an important element in a company that affects many areas of the company’s existence and its success. It enables individuals to understand how the company works and stimulates their behavior towards it. Hofstede (2015) called culture the “software of the mind,” i.e., the collective programming of the mind when examining the differences between cultures. The term culture includes everything that in some way leads people to behave and act in a certain way, both towards each other and towards their work and the physical world (Müller Dueholm and Axel Nielsen, 2013).

Organizational culture can be considered a key element that influences employee behavior and company performance, which is directed by company management (Melnyk et al., 2021). Employees should understand the corporate culture, otherwise, it will cause conflicts between the members of the organization, hinder the fulfillment of tasks, and threaten their results (Marcon et al., 2021).

Organizational culture in organizations strengthens human capital and creates and facilitates individual and collective learning. In this process, the manager has the role of supporter, motivator, stimulator, and promoter of new ideas (Marcon et al., 2021). Research group Febrina et al. (2021), using the SEM (search engine marketing) method, proved that organizational culture has a significant effect on employee job satisfaction, which is closely related to self-awareness, self-development, adherence to rules, or aggressiveness. In order to create an effective organizational culture, it is important to motivate workers by providing challenges, and opportunities for career growth, building effective communication between the leader and subordinates, and last, but not least, providing opportunities for a different way of thinking (Melnyk et al., 2021). Organizational culture encourages work teams to pursue common goals and fulfill the company’s mission (Barbaros, 2020). Using the potential of employees, their creativity, and ideas, leads to the improvement of the organizational culture. Thanks to this step, when the employees themselves participate in the adjustment and improvement of the organizational culture, it leads to an increase in work performance, work well-being, and also a reduction of stress (Eskiler et al., 2016).

Based on a questionnaire survey, Melnyk et al. (2021) states that the most important indicators of the formation of an effective organizational culture are: financial stability, health and well-being of employees, job security, the presence of a challenge at work, and a high level of motivation and career prospects. According to Prystupa-Rządca (2017), based on the symbolic interpretation paradigm, it follows that culture is most influenced by the vision of the founder and the industry in which the company operates. However, an evolving culture can also sometimes be a burden when the value an organization has adopted no longer fits the organization’s goals. The culture that has been internalized into the organization can no longer match the vision of the organization if the organization undergoes developments that change its vision and mission. A stagnant culture in an organization will become the reason for a decline in organizational performance (Tama, 2019). However, when it comes to creating and growing an organizational culture, it cannot be described solely in terms of members’ sense of comfort. There are many other factors to consider such as careful thinking and its effect on developing a culture that will have a good impact on the organization (Kundi, 2021).

Knowledge and changes in organizational culture are also closely related to the migration of people from different cultures and the ability of organizations to use the potential of skilled migrants and their motivation to settle in a new country and stay indefinitely (Farashah and Blomqusit, 2021). Building a positive organizational culture can help to engage different population groups and use their hidden potential (Bingöl et al., 2013). Organizations should invest in employee development programs that not only improve their technical skills but also develop their cultural intelligence. Cultural intelligence enables employees to adapt to different cultural environments, understand others’ perspectives, and build effective relationships across cultures. This ability is increasingly important as firms expand globally and operate with diverse stakeholders (Tulcanaza-Prieto et al., 2021).

Using the PLS-SEM method, Lee et al. (2018) demonstrated that organizational culture affects overall firm performance. Wahjoedi (2021) clarified the importance of the positive effect of quality leadership on organizational culture, work motivation, and employee performance, the positive effect of work motivation on organizational culture, and the positive effect of organizational culture on performance in the field under study (Paais and Pattiruhu, 2020).

One of the most frequently used methods of measuring organizational culture in relation to business performance is The Organizational Culture Assessment Instrument (OCAI) (Cameron and Quinn, 2011).

The OCAI method was developed and copyrighted in the 1980s and is one of the tools based on the Competing Values Framework (CVF). OCAI theory assumes the existence of two competing, independent core values, which are: (1) Internal focus with integration vs. external focus with differentiation; and (2) stability and control vs. flexibility and freedom of action (Flynn, 2022). CVF serves as a map, an organizational mechanism, a sensory tool, a source of new ideas, and a learning system (Bremer, 2018).

The OCAI questionnaire consists of six areas: (1) Dominant characteristics – characteristic features of the environment and prevailing atmosphere in the company. (2) Organizational leadership – what is considered leadership ability. (3) Management of employees – what is typical of the managerial style. (4) Organizational glue – how the company consolidates. (5) Strategic emphasis – what is emphasized and what is the goal of the company. (6) Success criteria – how success is defined in the company (Ližbetinová et al., 2016). Subsequently, each area is analyzed based on four statements, each statement corresponding to one of four types of organizational culture: clan culture (A), adhoc culture (B), market culture (C), and hierarchical culture (D).

Scientific studies address corporate culture through various combinations of relationships, comparing and in the context of other links such as information technology management in digitization (Sieber, 2019), implementation of HR activities and employer branding (Urbancová and Depoo, 2021), industry 4.0 and innovation (Mohelska and Sokolova, 2018), project portfolio management (Alexandrova, 2020) performance measurement and management system (Kotková Stříteská and Sein, 2021), strategic management, risk management and financial performance of the company (Syrová and Špička, 2023), CSR social responsibility (Mohelska and Sokolova, 2018), the Smart Factory concept (Gregar et al., 2021) and many others.

The OCAI method offers many advantages such as greater sophistication, easy comparability of the results at different levels of the company and state, trend prediction of the future state of the organizational culture, and other advantages.

## 3 Data and methods

The aim of the study is to evaluate the actual and desired state of the organizational culture of companies in the Czech Republic and subsequently to evaluate their dependence on the size of the companies. To fulfill the research objective, the universal tool OCAI is chosen, with the help of which it is possible to diagnose the dominant elements of the company’s orientation and to define the type, strength, and conformity of the prevailing culture. At the same time, this method shows the desired, i.e., preferred and expected culture. The desired culture in this case is the culture that respondents want to have in the future. This finding then serves as a starting point for changing the corporate culture. To achieve the main objective, the following research questions were set:

RQ1: What is the current and desirable state of organizational cultures in companies in the Czech Republic according to the individual researched areas?

RQ2: What is the difference in the actual evaluation of company culture in the Czech Republic according to company size?

RQ3: What is the difference in the required assessment of corporate culture in the Czech Republic according to company size?

The Organizational Culture Assessment Instrument (OCAI) questionnaire will be used as a research tool, which is the most cited, most used, and the most tested method in recent years. The subsequent assessment and analysis compare the actual and required state of corporate culture in six areas: (1) Dominant traits and characteristics, (2) Business management, (3) Management and collaboration style, (4) Cohesion and cohesion of the business, (5) Strategic focus, (6) Success criteria. Each area is divided into four categories and evaluated in percentages so that the sum of the individual categories is 100%. Individual evaluations represent the results of a questionnaire survey. The values of the individual categories are statistical variables, which are further analyzed using numerical characteristics, and graphs of their distribution and compared using statistical tests when examining their mutual dependence.

As part of the first research question, we compare the evaluation statuses of individual categories. The difference variables between the assessment of the actual and expected state are expressed by indices a, r, d (where the index “a” means the actual state, “r” means the desired one, and “d” means the difference. QB1A_d = QB1A_a – QB1A_r). To find that the mean ratings differ, we use the mean values of the different variables, whether the corresponding population means are significantly different, we use a one-sample *z*-test on the mean applied to the different variables. For more general comparisons, we will use one-tailed intervals for population means.

To evaluate research questions 2 and 3, individual scales of organizational cultures will be used as dependent variables, and tested for changes and differences based on company size (4 categories – micro-enterprise, small enterprise, medium-sized enterprise, and large enterprise). The OCAI questionnaire provides data on four types of organizational culture – clan culture (A), adhoc culture (B), market culture (C), and hierarchy culture (D), which are determined on the basis of percentage agreement with six questionnaire questions for each type of culture. The questionnaire ascertains the assessment of the actual state in the investigated enterprise and is supplemented by parallel questions where the desired state is evaluated. Thus, the results provide both of these scales for each type of culture and at the same time make it possible to examine the differences between the two assessments. Statistical analysis is performed in IBM SPSS 24 software using analysis of variance and *post hoc* tests (Tukey HSD). The level of significance was chosen to be 0.05.

The data will be obtained using a questionnaire survey, which will take place repeatedly in 2019, 2020, 2021. The Google Forms application will be used for the questionnaire survey and individual organizations will be contacted via e-mail based on the data in the commercial register of the Czech Republic. The data will then be processed in MS Excel and evaluated in IBM SPSS 24 software.

## 4 Results

The results were obtained from a total of 926 respondents, of which 431 in 2019, 299 in 2020, and the remaining 199 respondents in 2021 via online questionnaires. In the last 2 years, participation was lower due to the pandemic situation. In the group of respondents, company employees in managerial positions predominate (Table 1).

**Table 1:** Frequency of surveyed enterprises according to the size of the organization.

**Period**	**Micro**	**Small**	**Medium**	**Large**	**Total**
2019	38	121	140	132	431
2020	39	80	94	83	296
2021	18	43	66	72	199
Total	95	244	300	287	926

Source: Own processing.

### 4.1 Evaluation of the analysis of the dominant characteristics of the corporate culture of the Czech Republic

Table 2 shows the description of the individual categories, their designation as statistical variables, and the percentage rating of their average actual and average expected status. Clan culture (A) is rated the most for both states (31.7 and 37.7%) and the actual state is rated least for adhocratic culture (B) 21.5% and the desired state of hierarchical culture (D) 18.5%.

**Table 2:** Dominant features and characteristics of corporate culture.

Variable dominant traits and characteristics	Designation of variable	Average rating of the actual condition	Average rating of desired condition
(A) The company prefers a personal approach, it resembles a multi-member family. Workers are often in harmony with each other and have a lot in common.	QB1A	31.7%	37.1%
(B) The company is characterized by a dynamic and entrepreneurial atmosphere. Workers are willing to give in and take risks.	QB1B	21.5%	22.6%
(C) The company is strongly goal-oriented. The main interest is in completing work tasks. There is strong competitiveness and an orientation towards achieving a set goal.	QB1C	24.9%	21.8%
(D) The company is characterized by a strict approach, control and a fixed structure. Formal relations and management prevail here.	QB1D	21.9%	18.5%

Source: Own processing.

Table 3 contains sample means and standard deviations (in %), one-tailed *z*-test value of *p*s on means, and corresponding one-tailed confidence intervals for population means (in %) for each difference variable.

**Table 3:** Evaluation of difference variables for dominant traits.

Difference variable	Diameter	The value of *p* of the *z*-test	Standard deviation	One-sided confidence interval
QB1A_d	−5.40	4,22.10^−12^	23.71	(−∞; −3.87)
QB1B_d	−1.06	0,057	16.95	(−∞; 0.03)
QB1C_d	3.05	4,81.10^−6^	20.28	(1.74; ∞)
QB1D_d.	3.41	8,81.10^−7^	21.11	(2.05; ∞)

Source: Own processing.

The actual rating is most significantly smaller (by 5.4%) for clan culture (A), and most significantly larger (by 3.41%) for market culture (D). Whether the conclusion about the sample mean is significant, i.e., valid in general for the entire population of enterprises, we verify with a z-test about the mean of the difference variable Y. At the 5% level of significance, we test the null hypothesis that the population mean of the difference variable *Y* = 0 against the alternative hypothesis that the population mean difference variables Y ≠ 0.

If the value of *p* of the test is less than the 0.05 significance level, we reject the null hypothesis. In our case, the observed difference is significantly different for organizational cultures A, C, and D. This significance was not proven for the adhocratic culture (B). To calculate a confidence interval, we need the standard deviation. A one-sided confidence interval allows the *z*-test used to be generalized. We test the null hypothesis that the population means of the difference variable Y = k against the alternative hypothesis that the population means of the difference variable Y > k (right-sided test with a positive sample mean), or Y < k (left-tailed test for negative sample mean). If the constant k, in the null hypothesis, does not lie in the calculated one-sided confidence interval, we reject the null hypothesis. So in our case, the difference variable for clan culture (A) is significantly smaller than 0, −1, −2, −3, but this is no longer the case for k = −4. For hierarchical culture (D) the difference variable is significantly greater than 0, 1, 2, but this is no longer the case for *k* = 3.

### 4.2 Evaluation of the analysis of company management in the corporate culture of the Czech Republic

The corporate culture of the Czech Republic in the area of business management is evaluated in Table 4, where it is evident that the clan culture (A) is the most evaluated in both states and the market culture (C) the least. For cultures (clan, adhocratic, and hierarchical A, B, and D), the desired state is ranked higher, and for market culture (C), the actual state is ranked higher.

**Table 4:** Evaluation of company management in the corporate culture of the Czech Republic.

Business management	Designation of variable	Average rating of the actual condition	Average rating of desired condition
(A) Management takes the role of advisor, helper and protector.	QB2A	31.9%	35.6%
(B) Management is oriented towards gaining a lead/uniqueness in the market, towards innovation, and accepting a degree of risk.	QB2B	25.5%	26.3%
(C) Management generally favors aggressive pressure and goal-oriented interest.	QB2C	18.8%	13.5%
(D) Leadership is considered a demonstration of cooperative, organized, and smoothly functioning performance.	QB2D	23.8%	24.6%

Source: Own processing.

The actual rating is most significantly smaller (by 3.76%) for clan culture (A), and most significantly larger (by 5.37%) for market culture (C). In the case of business management, the observed difference is significantly different for clan (A) and market (C) cultures. For the adhocratic (B) and hierarchical D culture, this significance was not proven. So the difference variable for clan culture (A) is significantly smaller than 0, −1, −2, but this is no longer the case for *k* = −3. For market culture (C) the difference variable is significantly larger than 0, 1, 2, 3, 4, but this is no longer the case for *k* = 5, see Table 5.

**Table 5:** Evaluation of the different variables in business management.

Difference variable	Diameter	The value of *p* of the *z*-test	Standard deviation	One-sided confidence interval
QB2A_d	−3.76	1,06.10^−7^	21.82	(−∞; −2.35)
QB2B_d	−0.76	0,215	19.67	(−∞; 0.44)
QB2C_d	5.37	9,73.10^−17^	19.67	(4.10; ∞)
QB2D_d.	−0.85	0.200	20.19	(−∞; 0.45)

Source: Own processing.

### 4.3 Evaluation of the analysis of management style and cooperation in the corporate culture of the Czech Republic

The corporate culture of the Czech Republic in the area of management style and cooperation in the company is evaluated in Table 6, where it can be seen that the clan culture (A) is the most evaluated in both states (38.5 and 39.7%) and the market culture (C) the least, i.e., 12.8 and 11.4%. For clan (A) and adhocratic (B) cultures, the desired state is highly valued, and for market (C) and hierarchical (D) cultures, the actual state is highly valued.

**Table 6:** Evaluation of the style of management and cooperation in the corporate culture of the Czech Republic.

Management style variables and cooperation in the company	Designation of variable	Average rating of the actual condition	Average rating of desired condition
(A) Teamwork and cooperation of workers prevail	QB3A	38.5%	39.7%
(B) An individual approach prevails, with a high degree of risk-taking, innovation, freedom, and uniqueness	QB3B	17.4%	18.8%
(C) Ruthless competition with high demands and goals prevails	QB3C	12.8%	11.4%
(D) Emphasis on employee safety, work atmosphere, and stability of relationships in the work team prevails.	QB3D	31.3%	30.2%

Source: Own processing.

The actual rating is most significantly smaller (by 1.42%) in the adhocratic culture (B), and most significantly larger (by 1.41%) in the market culture (C). In the case of the style of management and cooperation in the company, the observed difference is significantly different for adhocratic (B) and market (C) cultures. For clan (A) and hierarchical (D) cultures, this significance was not demonstrated. So the difference variable for adhocratic culture (B) is significantly less than 0, but this is no longer the case for *k* = −1. For market culture (C) the difference variable is significantly greater than 0, but this is no longer the case for *k* = 1, see Table 7.

**Table 7:** Evaluation of differential variables for management and collaboration style.

Difference variable	Diameter	The value of *p* of the *z*-test	Standard deviation	One-sided confidence interval
QB3A_d	−1.15	0.103	21.51	(−∞; 0.23)
QB3B_d	−1.42	0.009	16.66	(−∞; −0.35)
QB3C_d	1.41	0.013	17.28	(0.29; ∞)
QB3D_d.	1.17	0.067	19.45	(−0.08; ∞)

Source: Own processing.

### 4.4 Evaluation of the analysis of cohesion and bonding in the company

The corporate culture of the Czech Republic in the area of cohesion and bonding in the company is evaluated in Table 8, where it is evident that the clan culture (A) is the most evaluated in both states (30.3 and 34.9%) and the adhocratic culture is the least evaluated (B) for the actual state 19.9% and hierarchical culture (D) for the desired state 19.7%. For clan (A) and adhocratic (B) cultures, the desired state is highly valued, and for market (C) and hierarchical (D) cultures, the actual state is highly valued. In the adhocratic culture (B), the differences in the evaluation of both conditions are small.

**Table 8:** Evaluation of cohesion and bonding in the corporate culture of the Czech Republic.

Cohesion and bonding in the company	Designation of variable	Average rating of the actual condition	Average rating of desired condition
(A) The company is bonded by the loyalty and mutual trust of employees and management.	QB4A	30.3%	34.9%
(B) The company connects commitment to innovation and development. Emphasis is placed on the highest level of development (technology, human resources).	QB4B	19.9%	21.5%
(C) The cohesive element is the emphasis on success and the achievement of set goals.	QB4C	26.5%	23.9%
(D) The binding element is formal rules and politics. Keeping the business running smoothly is important.	QB4D	23.3%	19.7%

Source: Own processing.

The actual rating is most significantly smaller (by 4.59%) for clan culture (A) and most significantly larger (by 3.56%) for hierarchical culture (D). In the case of cohesion and bonding in corporate culture, the observed difference is significantly different for all cultures. So in this case, the difference variable for clan culture (A) is significantly less than 0, 1, 2, 3, but it is no longer the case for *k* = −4. For the adhocratic culture (B), the difference variable is significantly less than 0, but this is no longer the case for *k* = −1. For the market culture (C), the difference variable is significantly greater than 0.1, but no longer for *k* = 2. For the hierarchical culture (D), the difference variable is significantly greater than 0.1, 2, but no longer for *k* = 3 (Table 9).

**Table 9:** Evaluation of difference variables for cohesion and bonding in the corporate culture of the Czech Republic.

Difference variable	Diameter	Difference variable	Standard deviation	One-sided confidence interval
QB4A_d	−4.59	4.68.10^−10^	22.40	(−∞; −3.14)
QB4B_d	−1.55	0,004	16.53	(−∞; −0.49)
QB4C_d	2.58	3.14.10^−5^	18.83	(1.36; ∞)
QB4D_d.	3.56	8.84.10^−9^	18.84	(2.35; ∞)

Source: Own processing.

### 4.5 Evaluation of the analysis of the company’s strategic orientation

The corporate culture of the Czech Republic in the area of the strategic focus of the company is evaluated in Table 10, where it can be seen that the clan culture (A) is the most evaluated in both conditions (29.7 and 32.9%) and in the year on the cultural market (C) least in both conditions (20.7 and 20.5%). In clan (A) and adhocratic (B) cultures, desired status is highly valued, and in market (C) and hierarchical (D) cultures, actual status is highly valued.

**Table 10:** Evaluation of the company’s strategic orientation.

Strategic orientation	Designation of variable	Average rating of the actual condition	Average rating of desired condition
(A) The company focuses on human relations, high trust, openness, and permanent cooperation.	QB5A	29.7%	32.9%
(B) The business focuses on acquiring new resources, creative challenges, and stimuli. The possibilities of new opportunities and innovations are valued.	QB5B	23.7%	25.1%
(C) The business focuses on competitive events and achievements. Achieving long-term goals and market position are dominant.	QB5C	20.7%	20.5%
(D) The business focuses on immutability and stability. Performance, control, and operability are important.	QB5D	26.0%	21.5%

Source: Own processing.

The actual assessment of the company’s strategic focus in the Czech Republic is most significantly smaller (by 3.28%) in the case of clan culture (A) and most significantly larger (by 4.47%) in the case of hierarchical culture (D). In the case of the company’s strategic orientation, the observed difference is significantly different for cultures A, B, and D. For market culture (C), no significant difference between the two evaluations was demonstrated. So in our case, the difference variable for clan culture (A) is significantly less than 0.1, but this is no longer the case for *k* = −2. For the adhocratic culture (B), the difference variable is significantly less than 0, but this is no longer the case for *k* = −1. For the market culture (C), the validity of the generalized test was not proven even for *k* = 0. For the hierarchical culture (D), the difference variable is significantly greater than 0, 1, 2, 3, but this is no longer true for *k* = 4, see Table 11.

**Table 11:** Evaluation of the different variables in the strategic focus of the company.

Difference variable	Diameter	Difference variable	Standard deviation	One-sided confidence interval
QB5A_d	−3.28	4.46.10^−6^	20.73	(−∞; −1.95)
QB5B_d	−1.40	0,011	16.72	(−∞; −0.32)
QB5C_d	0.21	0.742	19.13	(−1.03; ∞)
QB5D_d.	4.47	2.67.10^−11^	20.42	(3.16; ∞)

Source: Own processing.

### 4.6 Evaluation of the analysis of the criterion of success of the enterprise

The corporate culture of the Czech Republic in the field of business success criteria is evaluated in Table 12, where it can be seen that the clan culture (A) is rated the most in both states (28.2 and 31.6%) and the adhocratic culture (B) the least in both states (19.7 and 21.5%). For clan (A) and adhocratic (B) cultures, the desired state is highly valued, and for market (C) and hierarchical (D) cultures, the actual state is highly valued. For cultures B and C, the differences in the assessment of both conditions are small.

**Table 12:** Evaluation of enterprise success criteria.

Criteria for evaluating the company’s success	Designation of variable	Average rating of the actual condition	Average rating of desired condition
(A) The company defines success based on human resource development, teamwork, employee consent, and concern for its workers.	QB6A	28.2%	31.6%
(B) Success defines the way of disposing of unique or innovative products - the so-called leader of products and innovations.	QB6B	19.7%	21.5%
(C) Success is defined as winning over the competition and gaining a position in the market.	QB6C	25.6%	23.9%
(D) Success determines the performance of processes: reliable deliveries, mastered logistics, and low-cost production.	QB6D	26.5%	23.0%

Source: Own processing.

The actual assessment of the success criteria of the company in the Czech Republic is most significant (by 3.47%) in the case of clan culture (A), and most significantly greater (by 3.54%) in the case of a hierarchical culture (D). In this case, the observed difference is significantly different for all cultures. So the difference variable for clan culture (A) is significantly smaller than 0, 1, 2, but no longer for *k* = −3. For an adhocratic culture (B), the difference variable is significantly less than 0, but this is no longer the case for *k* = −1. For market culture (C), the difference variable is significantly greater than 0, but no longer for *k* = 1. For hierarchical culture (D), the difference variable is significantly greater than 0, 1, 2, but no longer for *k* = 3 (Table 13).

**Table 13:** Evaluation of the difference variables for the success criterion.

Difference variable	Diameter	Difference variable	Standard deviation	One-sided confidence interval
QB6A_d	−3.47	1.36.10^−7^	20.01	(−∞; −2.18)
QB6B_d	−1.75	0.0012	16.45	(−∞; −0.69)
QB6C_d	1.68	0.0077	19.12	(0.44; ∞)
QB6D_d.	3.54	1.31.10^−9^	17.77	(2.40; ∞)

Source: Own processing.

If we compare the required evaluation of individual cultures with each other in individual areas, the following results can be arrived at. The surveyed respondents wish for a friendly and family environment where mutual understanding prevails. On the contrary, it does not suit an environment where there is a fixed structure and strict control.

In terms of leadership, the leader should be like a counselor, helper, and defender, strongly rejecting aggressive pressure and a goal-oriented leader. This can be read from the significant difference of 22.1% between these cultures (A and C).

When asked which management method they prefer, 39.7% of respondents answered teamwork, but only 11.4% of respondents want a competitive approach with high goal requirements.

The unifying element of a company in the Czech Republic is loyalty and mutual trust between employees and management, not formal rules and company policy.

When it comes to the company’s strategic orientation, companies in the Czech Republic want to focus more on interpersonal relationships, trust openness, and cooperation than on success and defending a dominant position in the market. In this case, the difference is 12.4%. The success of the enterprise depends more on the development of human resources than on unique innovative products.

If we add an average for each row in the six regions, then we get the overall average of the respective culture. For example, adding the first row (QB1A, QB2A, QB3A, QB4A, QB5A, and QB6A) gives the clan culture rating (A).

## 5 Discussion

In this section, we will try to answer the research questions.

### 5.1 What is the actual and desired state of organizational cultures in companies in the Czech Republic according to the individual researched areas?

The six individual dimensions were examined in detail in the previous results section, so here we summarize the overall result. Enterprises in the Czech Republic have a mix of organizational cultures in the following order: (1) clan culture (31.72%), (2) hierarchical culture (25.46%), (3) market culture (21.5%), and (4) adhocratic culture (21.28%).

Clan culture, which is most preferred, got its name based on its resemblance to a family structure. Employees are encouraged to work in teams and help each other. Management encourages employees to share ideas, secure their loyalty, and create a friendly place to work. It also pays attention to a partnership approach to customers. Success is determined by internal workplace climate and employee care (Cameron and Quinn, 2006). Interestingly, a hierarchical culture in which a lot of emphasis is placed on structure came in second place. In this culture, there are precisely given procedures and instructions for everything, and excessive formality is evident. For an organization with a hierarchical culture, it is most important that smooth operations are maintained and the goal is to achieve stability and as much efficiency as possible (Cameron and Quinn, 2006). From the results found, it can be inferred that organizational cultures in Czech companies have a more internal orientation and are more oriented towards integration.

We cannot sufficiently compare the results of other studies with ours because we have not yet found a study conducted across all disciplines. Nevertheless, we find similar results in some studies. E.g. clan and hierarchical organizational culture are dominant in the Slovenian logistics sector (Čuček and Mlaker Kač, 2020), clan culture in the Romanian private sector (Dobrin et al., 2021), clan and hierarchical culture in the Portuguese healthcare sector (Albino et al., 2022) or in Swedish organizations (Farashah and Blomqusit, 2021).

Regarding the desired state of culture, the order changes in third and fourth place: (1) clan (35.3%), (2) hierarchical (22.91%), (3) adhocratic (22.63%), and (4) market culture (19.17%).

An adhocratic culture that is more desired than actually practiced focuses on innovation, new product development, and rapid growth. An organization with this type of culture is adaptable and creative but operates in an environment of uncertainty and risk. Success is measured by the production of new and unique products (Cameron and Quinn, 2006). The least required is a market culture, which represents an external focus in which the emphasis is on customers, suppliers, unions, etc. The focus is on profit, productivity, and competitiveness. This type uses an aggressive strategy to achieve its goals, the only way it can succeed in an external hostile environment. Success is determined by market share (Cameron and Quinn, 2006). From the results of the research, we can conclude that in the Czech Republic, there is no big difference between preferred and applied organizational cultures. The difference exists only in less preferred cultures, where market culture is used more, which brings less differentiation and more stability compared to *ad hoc* culture. However, the development of the market environment and competition can influence individual companies to start striving more for the implementation of an *ad hoc* culture.

Similar results were recorded in comparative research in the Czech Republic, Slovakia, and the People’s Republic of China, where similarities in corporate cultures and a preference for clan corporate culture can be seen. In the Czech Republic, loyalty and mutual trust between employees and management are considered essential, not formal rules and company policy. Lorincová et al. (2020) demonstrated that the opposite could be the Russian Federation, where employees prefer market and hierarchical corporate cultures (Lorincová et al., 2020).

### 5.2 What is the difference in the actual assessment of the corporate culture of the Czech Republic according to the size of the company?

The average values in the individual scales of organizational culture according to the size of the company are shown in Table 14. The variance analysis shows a difference in the actual value of clan culture *F*(df = 3,922) = 37.39, *p* < 0.01, market culture *F*(df = 3,922) = 10.74, *p* < 0.01, and hierarchy culture *F*(df = 3,922) = 17.41, *p* < 0.01.

**Table 14:** Average values of the OCAI questionnaire by company size.

		Micro (0–9)			Small (10–49)			Medium (50–249)			Large (250+)		
		*Act*	*Prefer*	*Dif*	*Act*	*Prefer*	*Dif*	*Act*	*Prefer*	*Dif*	*Act*	*Prefer*	*Dif*
**Clan culture**	*m*	41.27	40.62	0.65	36.55	38.78	−2.23	30.32	33.91	−3.60	25.84	32.04	−6.20
	*sd*	*17.75*	*17.23*		*17.11*	*17.04*		*13.07*	*14.60*		*13.56*	*14.94*	
**Adhocracy culture**	*m*	19.60	21.65	−2.06	20.88	21.57	−0.69	21.99	23.12	−1.14	21.49	23.31	−1.82
	*sd*	*8.67*	*8.34*		*8.48*	*9.09*		*7.93*	*7.90*		*8.51*	*8.49*	
**Market culture**	m	17.21	16.60	0.62	20.03	18.48	1.55	22.00	19.71	2.28	23.79	20.02	3.77
	*sd*	*10.90*	*9.26*		*10.91*	*10.52*		*10.14*	*9.12*		*11.74*	*9.78*	
**Hierarchy culture**	m	21.93	21.13	0.79	22.54	21.17	1.37	25.70	23.25	2.45	28.88	24.64	4.24
	*sd*	*9.34*	*10.36*		*9.75*	*9.14*		*10.73*	*10.95*		*13.41*	*10.82*	

Source: Own processing.

*Post hoc* tests (Tukey HSD, *p* < 0.05) were performed between individual business types, the results of which are included in Table 14. In the evaluation of the actual situation in the clan culture scale, a statistically significant trend was found, where the smaller the type of business, the higher the value in the clan culture scale (250+ < 50–249 < 9–49 < 0–9). In market culture, the difference between a micro-enterprise and a medium and large enterprise (0–9 < 50–249, 250+) and between a small enterprise and a large enterprise (10–49 < 250+) was highlighted. In hierarchy culture, a difference was found compared to medium and large enterprises in micro enterprises (0–9 < 50–249, 250+) and small enterprises (10–49 < 50–249, 250+). At the same time, a difference was also found between medium and large enterprises (50–249 < 250+).

A statistically significant trend was found for the actual state of the clan culture, where the smaller the enterprise, the higher the rating of this culture. Differences in market and hierarchical culture can also be read from the analysis of variance results. From these results, it can be concluded that the larger the company, the more it prefers a hierarchical culture over a market one. The results of the *post hoc* test show differences in market culture between micro-enterprises and medium and large enterprises, and further between small and large enterprises. Where the results show that the bigger the company, the more market culture is preferred and used. A difference in hierarchical culture between medium and large enterprises was found in micro-enterprises and small enterprises, where it is confirmed that with the growth of the enterprise, there is a greater preference and use of hierarchical culture.

### 5.3 What is the difference in the required assessment of the corporate culture of the Czech Republic according to the size of the company?

The results of the analysis of variance show differences in all four cultures. A statistically significant trend was found for the desired state of clan culture, where the larger the company, the more it prefers clan culture. With the help of a *post hoc* test, it was found that there are differences in market and adhocratic culture between micro-enterprises and medium and large enterprises. Furthermore, a difference was found in hierarchical culture compared to a large enterprise in micro-enterprises and small enterprises.

A statistically significant difference in the assessment of organizational culture depending on the size of the organization was also demonstrated by Sokolova et al. (2019), which used the OCI method to diagnose organizational culture. The authors came to the same conclusion that a statistically significant difference was observed in the evaluation of the organizational culture index, especially depending on the size of the organization in the Czech Republic (Sokolova et al., 2019).

## 6 Conclusion

Organizational culture has a great influence on the image and identity of the organization. It is a set of habits, values, norms, and behavior that is created in an organization and affects practically all aspects of the organization’s activities. If an organization focuses on creating a positive culture with clear values, promoting professional employee behavior, and encouraging innovation, it can help strengthen the organization’s image and identity.

Companies try to gain every possible advantage over their competitors and this very often means implementing creative management approaches. The introduction of these cutting-edge methods alone does not lead to improved business performance or other benefits, and therefore the introduction must be supported by a positive organizational culture. Here, organizational culture acts as a catalyst for implemented changes.

The aim of this work was to identify differences in the perception of the preferred and current level of organizational culture of companies in the Czech Republic and subsequently to evaluate their dependence on the size of organizations using the OCAI diagnostic method.

The results of the study show that clan culture prevails in the Czech Republic, which was dominant in all six dimensions. A statistically significant trend was found for the actual state of clan culture, where the smaller the enterprise, the higher the value of this culture. The results also reveal that the larger the company, the more it would like to implement clan culture, but in reality, other types of cultures prevail here.

This work can serve as a comparative basis of organizational culture with another national culture. It also shows the trend of the development of culture in individual dimensions in the Czech Republic, so that managers understand the necessity of changing corporate culture and help popularize this issue.

After all, there is no ultimate “best” organizational culture prescribed by a competing value framework. This model is descriptive. In a certain domain or market, one type of culture may suit better than another, and this must be decided by the organization itself.

## Data availability statement

The raw data supporting the conclusions of this article will be made available by the authors, without undue reservation.

## Ethics statement

Ethical review and approval was not required for the study on human participants in accordance with the local legislation and institutional requirements. Written informed consent from the patients/participants or patients/participants legal guardian/next of kin was not required to participate in this study in accordance with the national legislation and the institutional requirements.

## Author contributions

MB: Writing – original draft, Writing – review & editing. TJ: Data curation, Methodology, Writing – original draft.
